# Consensus Design of an Evolved High-Redox Potential Laccase

**DOI:** 10.3389/fbioe.2020.00354

**Published:** 2020-05-06

**Authors:** Bernardo J. Gomez-Fernandez, Valeria A. Risso, Jose M. Sanchez-Ruiz, Miguel Alcalde

**Affiliations:** ^1^Department of Biocatalysis, Institute of Catalysis, CSIC, Madrid, Spain; ^2^Facultad de Ciencias, Departamento de Química Física, Universidad de Granada, Granada, Spain

**Keywords:** consensus design, high-redox potential laccase, ancestor mutation, thermostability, activity

## Abstract

Among the broad repertory of protein engineering methods that set out to improve stability, consensus design has proved to be a powerful strategy to stabilize enzymes without compromising their catalytic activity. Here, we have applied an in-house consensus method to stabilize a laboratory evolved high-redox potential laccase. Multiple sequence alignments were carried out and computationally refined by applying relative entropy and mutual information thresholds. Through this approach, an ensemble of 20 consensus mutations were identified, 18 of which were consensus/ancestral mutations. The set of consensus variants was produced in *Saccharomyces cerevisiae* and analyzed individually, while site directed recombination of the best mutations did not produce positive epistasis. The best single variant carried the consensus-ancestral A240G mutation in the neighborhood of the T2/T3 copper cluster, which dramatically improved thermostability, kinetic parameters and secretion.

## Introduction

The distribution of amino acids in multiple sequence alignments (MSAs) of homologous proteins reveals the most conserved positions in the different sequences (Lehmann et al., 2000; Lehmann and Wyss, 2001). The majority of these residues are considered to be stabilizing since they help to withstand the demands of adaptations that occur in the course of natural evolution. The identification and insertion of such consensus mutations in a protein template is known as consensus design, and it is thought to help stabilize enzymes while bypassing the unwanted trade-off between activity and stability (Porebski and Buckle, 2016; Siddiqui, 2017). Compared with other strategies to improve thermostability [e.g., directed evolution, rational rigidification of flexible loops, computational methods like FRESCO, SCHEMA or PROSS, ancestral libraries or protein resurrection (Cole and Gaucher, 2011; Nestl and Hauer, 2014; Magliery, 2015; Reetz, 2017)], consensus design has some appealing advantages that make it a good choice to be applied individually or in combination with the aforementioned approaches. Indeed, consensus mutagenesis establishes an optimum balance between library size and the required knowledge of the protein, which allows smart and functionally enriched mutant libraries to be generated from MSAs, while streamlining resources and research efforts (Lehmann et al., 2000, 2002; Jackel et al., 2010).

Fungal laccases (EC 1.10.3.2) are multi copper oxidases of broad biotechnological interest, including among their main uses several sectors such as food, textiles, pulp and paper, pharma, biofuels, cosmetics and bioremediation (Alcalde, 2007; Rodgers et al., 2010; Mate and Alcalde, 2017). Structurally organized into three cupredoxin domains, laccase catalysis is governed by the T1Cu site where the substrate binds, and the T2/T3 trinuclear copper cluster located 12 Å away that is involved in the conversion of molecular oxygen to water (Jones and Solomon, 2015). In this context, high-redox potential laccases (HRPLs) from white-rot fungi are of particular interest as they have outstanding oxidative capabilities that are dependent on both a high-redox potential at the T1Cu site and a relaxed substrate binding mode (Rodgers et al., 2010; Mate and Alcalde, 2015; Mateljak et al., 2019a). While we are witnessing a blossom of HRPL engineering in an attempt to cover different industrial needs, including laboratory evolution campaigns to retain activity in non-conventional media or to increase activities on different compounds, thermostability has been largely overlooked during such efforts (Hildén et al., 2009; Mate and Alcalde, 2015). However, in recent years we have paid special attention to this issue by embarking on SCHEMA recombination and structure-guided evolution experiments (Mateljak et al., 2019b; Vicente et al., 2020).

In this work, we have harnessed consensus design to enhance the stability of a HRPL variant (the OB-1 mutant), a product of several rounds of directed evolution to improve functional expression in yeast (Mate et al., 2010). We first constructed three MSAs that were filtered by relative entropy (RE) and mutual information (MI) thresholds (Sullivan et al., 2012; Durani and Magliery, 2013). From this *in silico* analysis, 20 single consensus mutants were selected, characterized and aligned with the inferred ancestral node of all Basidiomycota laccases to identify consensus/ancestral mutations. The most promising mutations were subjected to site-directed recombination *in vivo* to search for positive epistatic combinations, and the final mutant was characterized biochemically.

## Materials and Methods

### Strains and Chemicals

All chemicals were reagent-grade purity. ABTS (2,2′-azino-bis(3-ethylbenzothiazoline-6-sulphonic acid), 2.6-dimethoxyphenol (DMP), sinapic acid, guaiacol, potassium octacyanomolybdate (IV) (K_4_[Mo(CN)_8_]), violuric acid and the Yeast Transformation Kit were purchased from Sigma (St. Louis, MO, United States). Zymoprep Yeast Plasmid Miniprep and Zymoclean Gel DNA Recovery Kit were from Zymo Research (Orange, CA, United States). NucleoSpin Plasmid kit was purchased from Macherey-Nagel (Düren, Germany). The uracil independent and ampicillin resistance shuttle vector pJRoC30 was obtained from the California Institute of Technology (Caltech, CA, United States). The protease deficient *S. cerevisiae* strain BJ5465 (α ura3-52 trp1 leu2Δ1 his3Δ200 pep4:HIS3 prb1Δ1.6R can1 GAL) was from LGC Promochem (Barcelona, Spain). The *Escherichia coli* strain XL2-Blue competent cells and Pfu DNA Polymerase were obtained from Agilent Technologies (Santa Clara, CA, United States). Restriction endonucleases *Bam*HI and *Xho*I were purchased from New England Biolabs (Ipswich, MA, United States). Oligonucleotide primers were acquired from Isogen Life Science (Barcelona, Spain).

### Culture Media

Minimal medium SC contained 100 mL of 6.7% (w/v) sterile yeast nitrogen base, 100 mL of a 19.2 g/L sterile yeast synthetic drop-out medium supplement without uracil, 100 mL of sterile 20% (w/v) raffinose, 700 mL of sterile double-distilled H_2_O (sddH_2_O), and 1 mL of chloramphenicol at 25 g/L. SC plates contained 100 mL 6.7% filtered yeast nitrogen base, 100 mL 19.2 g/L filtered yeast synthetic drop-out medium supplement without uracil, 20 g autoclaved bacto agar, 100 mL 20% filtered glucose, 1 mL 25 g/L filtered chloramphenicol and ddH_2_O to 1 L. YP medium contained 10 g of yeast extract, 20 g of peptone, and sddH_2_O to 650 mL, whereas YPD medium also contained 20% (w/v) glucose. Laccase selective expression medium (SEM) for 96 well plate contained 10 mL of 6.7% (w/v) sterile yeast nitrogen base, 10 mL of 19.2 g/L sterile yeast synthetic drop-out medium supplement without uracil, 10 mL of sterile 20% (w/v) galactose, 6.7 mL of sterile 1 M KH_2_PO_4_ (pH 6.0) buffer, 3.16 mL of absolute ethanol, 0.1 mL of a sterile 1 M CuSO_4_ solution, 0.1 mL of sterile chloramphenicol at 25 g/L, and sddH_2_O to 60 mL. Laccase liquid expression media for flask contained the same components as SEM except for 55.5 mL of 2X YP instead of yeast nitrogen base and yeast synthetic drop-out medium supplement without uracil and 0.2 mL of 1 M CuSO_4_ instead of 0.1 mL, for the same final volume. Luria broth (LB) medium contained 10 g of sodium chloride, 5 g of yeast extract, 10 g of peptone, 1 mL of sterile ampicillin at 100 mg/mL, and sddH_2_O to 1 L. LB plates contained 10 g of sodium chloride, 5 g of yeast extract, 10 g of peptone, 15 g of agar, 1 mL of sterile ampicillin at 100 mg/mL, and sddH_2_O to 1 L.

### Consensus Design

#### Multiple Sequence Alignments

PM1Lac sequence was retrieved from the National Center for Biotechnology Information (NCBI) (NCBI Resource Coordinators, 2018), entry CAA78144.1. Three different MSAs were created; MSA1 sequences were obtained from NCBI database upon BLASTp search (Altschul et al., 1997), using the following parameters: PM1Lac as query sequence, non-redundant protein sequences, maximum of 250 sequences to display and a threshold of percentage identity between 35 and 98%; MSA2 sequences were retrieved following the same steps as for MSA1 sequences but establishing a maximum of 1,000 sequences to display; For MSA3 sequences, all the sequences of basidiomycete laccases were recovered from the Laccase and Multicopper Oxidase Engineering Database (LccED) (Sirim et al., 2011). All sequences including terms such as “hypothetical,” “predicted” or “putative” were discarded, as well of those sequences coming from insects or ascomycetes, resulting in a set of 235 for MSA1, 819 for MSA2 and 477 for MSA3. PM1Lac sequence was added to the three groups of sequences and then they were aligned independently using MUSCLE (Edgar, 2004) generating the three MSAs as a result.

#### Data Mining From the MSAs

The three MSAs were imported into Microsoft Excel to refine them and to calculate RE and MI metrics for each position as well as a MI noise from a randomized version of the alignment as described elsewhere (Durani and Magliery, 2013) with minor modifications. The resulting information for all positions was compared with OB-1 sequence and after analyzing them, 20 substitutions were selected for the *in vitro* experiments.

### Alignment, Phylogeny and Ancestral Sequence Reconstruction

Fungal laccases homologs were retrieved from the April 2015 release of the complete genomes database available at National Center for Biotechnology Information (NCBI) (NCBI Resource Coordinators, 2018). All sequences including terms such as “hypothetical,” “predicted” or “putative” were discarded, as well of those sequences coming from insects or ascomycetes, resulting in a set of 120. The sequences were aligned using MUSCLE (Edgar, 2004) and a distance matrix was then generated using one minus the sequence similarity as the parameter to assess the evolutionary distance between two sequences. The distribution for the calculated distances revealed two different groups and we found that the major clade of Basidiomycota, the Agaricomycotina, class Agaricomycetes was overrepresented over the rest. For phylogenetic analysis, we employed the 87 sequences closest to the query since MrBayes efficiency relies strongly on sequence set size. Zygomycota and ascomycota sequences were also included as outgroups to generate a rooted tree making a total of 89 sequences. The tree topology and the branch lengths of the trees were estimated from these sequences using the Bayesian method implemented in version MrBayes 3.1.2^1^. This analysis used the Jones substitution model and two independent Markov-chain Monte Carlo runs, each with four chains, and it was performed for 2,113,000 generations to ensure adequate convergence (0.04). The nodes obtained in the tree had posterior probabilities higher than 0.8, with very few exceptions. We targeted the nodes 95 with a probability close to unity (Supplementary Figure S1). The sequence reconstruction was performed using PAML version 4.4e^2^ and with the WAG evolutionary model^3^.

### Library of Consensus Variants

#### General Aspects

PCR fragments were cleaned, concentrated and loaded onto a low melting point preparative agarose gel (Bio-Rad, Hercules, CA, United States), and then purified using the Zymoclean gel DNA recovery kit (Zymo Research, Orange, CA, United States). PCR products were then mixed with the previously linearized vector pJRoC30 with *Xho*I and *Bam*HI (at a PCR product/linearized plasmid ratio of 4:1) and transformed into competent *S. cerevisiae* cells. The whole gene was reassembled *in vivo* by transformation into *S. cerevisiae* via the design of ∼40-bp overhangs flanking each recombination area.

#### Construction of Single Variants by Site Directed Mutagenesis

The 20 single mutants from the consensus analysis were designed by *In vivo* Overlap Extension (IVOE) (Alcalde, 2010). OB-1 mutant was used as template and two independent PCRs (PCR1 and PCR2) were performed for each of the 20 constructions. Reaction mixtures were prepared in a final volume of 50 μL containing DNA template (0.2 ng/μL), 0.25 μM of each primer, 0.3 mM dNTPs (0.075 mM each), 3% (v:v) dimethylsulfoxide (DMSO) and 0.05 U/μL Pfu DNA polymerase. The primers used for each clone design are provided in Supplementary Table S1. PCRs were performed in a thermocycler (MyCycler, Bio-Rad,Hercules, CA, United States) and parameters were: 94°C for 2 min (1 cycle); 94°C for 45 s, 50°C for 30 s, 72°C for 1 min (30 cycles); and 72°C for 10 min (1 cycle). PCR products were mixed in equimolar amounts, 200 ng PCR1 product and 200 ng PCR2 product and transformed with linearized plasmid (100 ng) into chemically competent cells. PCR products were transformed independently in pairs, so just one position was mutated per transformation. Transformed cells were plated on SC plates and incubated for 3 days at 30°C. Colonies containing the whole autonomously replicating vector were picked and after analyzed 16 clones per mutation, the selected plasmids were extracted and sequenced.

### Site Directed Recombination Library

DooKu variant (A240G) was used as template and mutations M52L, T179G, L217M, V219I, Q230L and D250N were targeted for site directed recombination (SDR) *in vivo*. PCR1 used oligo sense RMLN and oligo antisense *52Rev* (containing position 52). PCR2 was performed with oligo sense *52For* (containing position 52) and a mix of oligos antisense *179wRev* and *179mutRev* (containing position 179). PCR3 used oligos sense *179wFor* and *179mutFor* (containing position 179) and oligo antisense *219Rev* (containing positions 217, 219, and 230). PCR4 used oligo sense *219For* (containing positions 217, 219, and 230) and oligo antisense *250Rev* (containing position 250). PCR5 was performed with oligo sense *250For* (containing position 250) and oligo antisense RMLC (Supplementary Figure S2A and Supplementary Table S2). For the *in vivo* assembly of the whole gene, the fragments were designed with overhangs of ∼40 bp flanking them. Reaction mixtures were prepared in a final volume of 50 μL containing DNA template (0.2 ng/μL), 0.25 μM of each primer, 0.3 mM dNTPs (0.075 mM each), 3% (v:v) dimethylsulfoxide (DMSO) and 0.05 U/μL Pfu DNA polymerase. The amplification parameters were 94°C for 2 min (1 cycle); 94°C for 45 s, 50°C for 30 s, 72°C for 2 min (30 cycles); and 72°C for 10 min (1 cycle). PCR products were mixed in equimolar amounts, 240 ng from each of the five PCRs products and transformed with linearized plasmid (300 ng) into chemically competent cells. Transformed cells were plated on SC plates and incubated for 3 days at 30°C. Colonies containing the whole autonomously replicating vector were submitted to high-throughput screening for thermostability.

### High-Throughput Screening

Individual clones were picked and inoculated in sterile 96-well plates (Greiner Bio-One, GmbH, Germany), referred to as master plates, containing 200 μL of SEM per well. In each plate, column number 6 was inoculated with the parent type, and one well (H1-control) was inoculated with *S. cerevisiae* transformed with pJRoC30-AAO plasmid (aryl-alcohol oxidase without activity toward ABTS). Plates were sealed with parafilm to prevent evaporation and incubated at 30°C, 220 rpm and 80% relative humidity in a humidity shaker (Minitron, Infors, Switzerland) for 3 days. The master plates were centrifuged (Eppendorf 5810R centrifuge, Germany) for 10 min at 2,500 *g* and 4°C. Aliquots of the supernatants were transferred from the master plates to two replica plates with the aid of a liquid handler robotic station Freedom EVO (Tecan, Switzerland), 50 μL of mixture to a thermocycler plate (Multiply PCR plate without skirt, neutral, Sarstedt, Germany) and 20 μL to the initial activity plate. Thermocycler plates were sealed with thermoresistant film (Deltalab, Spain) and incubated at the corresponding temperature using a thermocycler (MyCycler, Bio-Rad). Incubation took place for 10 min (so that the assessed activity was reduced by two-thirds of the initial activity). Afterward, thermocycler plates were placed on ice for 10 min and further incubated for 5 min at room temperature. 20 μL of supernatants were transferred from both thermocycler and initial activity plates to new plates to estimate the initial activities and residual activities values by adding 180 μL of 100 mM citrate phosphate buffer pH 4.0 containing 1 mM ABTS. Plates were stirred briefly and the absorption at 418 nm (ε_418_ABTS^∙+^ = 36,000 M^–1^ cm^–1^) was followed in kinetic mode in the plate reader (SPECTRAMax Plus 384, Molecular Devices, Sunnyvale, CA, United States). The same experiment was performed for both the initial activity plate and residual activity plate. The values were normalized against the parent type of the corresponding plate and selected variants came from the ratio between residual activities and initial activities values. To rule out false positives, three consecutive rescreenings were carried out according to the protocol previously reported with some modifications (Mate et al., 2010).

#### First Re-screening

Aliquots of 5 μL of the best clones were removed from the master plates to inoculate 50 μL of minimal media in new 96-well plates. Columns 1 and 12 (rows A and H) were not used to prevent the appearance of false positives. After a 24 h incubation at 30°C and 225 rpm, 5 μL was transferred to the adjacent wells and further incubated for 24 h. Finally, 160 μL of expression medium was added and the plates were incubated for 24 h. Accordingly, each mutant was grown in 4 wells. The parental types were subjected to the same procedure (lane D, wells 7-11) and the plates were assessed using the same protocols for the screening described above.

#### Second Re-screening

An aliquot from the wells with the best clones of the first rescreening was inoculated in 3 mL of YPD and incubated at 30°C and 225 rpm for 24 h. The plasmids from these cultures were extracted (Zymoprep yeast plasmid miniprep kit, Zymo Research). As the product of the zymoprep was very impure and the concentration of DNA extracted was very low, the shuttle vectors were transformed into super-competent *E. coli* cells (XL2-Blue, Stratagene) and plated onto LB-amp plates. Single colonies were picked and used to inoculate 5 mL LB-amp media and they were grown overnight at 37°C and 225 rpm. The plasmids were then extracted (NucleoSpin^®^ Plasmid kit, Macherey-Nagel, Germany) and *S. cerevisiae* was transformed with plasmids from the best mutants as well as with the parental type. Five colonies for each mutant were picked and rescreened as described above.

#### Third Re-screening

Fresh transformants of selected mutants and of the parental types were cultivated (10 mL) in a 100 mL flask for laccase production. The supernatants were subjected to a thermostability assay to accurately estimate their half-life of inactivation time (*t*_1/2_) values using 96/384 well gradient thermocyclers as described in the section of biochemical characterization.

### Laccase Production and Purification

A single colony from the *S. cerevisiae* clone containing the parental or mutant laccase gene was picked from a SC plate, inoculated in 10 mL of minimal medium and incubated for 48 h at 30°C and 225 rpm. An aliquot of cells was removed and inoculated into a final volume of 50 mL of minimal medium in a 500 mL flask (optical density, OD_600_ = 0.25). Incubation proceeded until two growth phases were completed (6–8 h) and then, 450 mL of expression medium was inoculated with the 50 mL preculture in a 2L flask (OD_600_ = 0.1). After incubating for 72 h at 30°C and 225 rpm (maximal laccase activity; OD_600_ = 28–30), the cells were separated by centrifugation for 20 min at 8,000 rpm (4°C) (Avantin J-E Centrifuge, Beckman Coulter, Fullerton, CA, United States) and the supernatant was double-filtered (through both a glass and then nitrocellulose membrane of 0.45 μm pore size). The crude extract was first submitted to a fractional precipitation with ammonium sulfate at 55% (first cut) and the pellet was then removed before the supernatant was subjected to 75% ammonium sulfate precipitation (second cut). The final pellet was recovered in 20 mM Bis Tris buffer pH 6.2 (buffer A) and the sample was filtered and loaded onto the fast protein liquid chromatography (FPLC) equipment (ÄKTA purifier, GE Healthcare, WI, United States) coupled with a strong anionic exchange column (HiTraP QFF, Amersham Bioscience) pre-equilibrated with buffer A. The proteins were eluted with a linear gradient from 0 to 1 M of NaCl in two phases at a flow rate of 1 mL/min: from 0 to 45% over 50 min and from 45 to 100% over 10 min. Fractions with laccase activity were pooled, concentrated, dialyzed against buffer A and loaded onto a 10 μm high resolution anion exchange column Biosuite Q (Waters, MA, United States) preequilibrated with buffer A. The proteins were eluted with a linear gradient from 0 to 1 M of NaCl at a flow rate of 1 mL/min in two phases: from 5 to 16% in 30 min and from 16 to 100% in 10 min. The fractions with laccase activity were pooled, dialyzed against 10 mM Bis Tris buffer pH 7.0, concentrated and stored at 4°C. The purified laccases were analyzed by SDS-polyacrylamide gel electrophoresis (SDS-PAGE). All protein concentrations were determined using the Bio-Rad protein reagent and bovine serum albumin as a standard.

### Biochemical Characterization

#### Half-Life (*t*_1/2_)

Appropriate dilutions of enzyme samples were prepared with the help of the liquid handler robotic station in such a way that aliquots of 20 μL give rise to a linear response in kinetic mode. 50 μL were used for each point in the incubation. A fixed temperature of 70°C was established for consensus mutations and 75°C for the SDR variants. Samples were removed at different times (0, 5, 15, 25, 35, 55, 75, 95, and 120 min) from the thermocycler (MyCycler, Bio-Rad) and chilled out on ice for 10 min. After that, samples of 20 μL were removed and incubated at room temperature for 5 min. Finally, samples were subjected to the same ABTS-based colorimetric assay described above for the screening. Thermostabilities values were deduced from the ratio between the residual activities incubated at different temperature points and the initial activity at room temperature.

#### pH Activity Profile

Appropriate dilutions of enzyme samples were prepared with the help of the liquid handler robotic station in such a way that aliquots of 20 μL give rise to a linear response in kinetic mode. Vessels containing 100 mM Britton and Robinson buffer with 1 mM of ABTS or 3 mM of DMP, were prepared at pH values of 2.0, 3.0, 4.0, 5.0, 6.0, 7.0, 8.0, and 9.0. The assay started when the different reactions mixtures of ABTS or DMP was added to each well. The activities were measured in triplicate in kinetic mode, and the relative activity (in percent) is based on the maximum activity for each variant in the assay.

#### pH Stability

Enzyme samples were incubated at different times over a range of pH values in 100 mM Britton and Robinson buffer (2.0, 3.0, 4.0, 5.0, 6.0, 7.0, 8.0, and 9.0). Samples were removed at different times (0, 4, 24, 48, 72, and 144 h) and subjected to the same ABTS-based colorimetric assay described above for the screening. The activities were measured in triplicate in kinetic mode, and the residual activity was deduced from the activity obtained at each time.

#### Estimation of Initial Activity Rates

Appropriate dilutions of enzyme samples were prepared with the help of the liquid handler robotic station in such a way that aliquots of 20 μL give rise to a linear response in kinetic mode. Initial activities for ABTS, sinapic acid, violuric acid and K_4_[Mo(CN)_8_] were estimated in sodium phosphate/citrate buffer (pH 4.0, 100 mM). Reactions were performed in triplicate, and substrate oxidations were followed at the corresponding maximum wavelengths of each reaction product (ε_418_ABTS^∙+^ = 36,000 M^–1^ cm^–1^; ε_512_ Sinapic acid = 14,066 M^–1^ cm^–1^; ε_515_ Violuric acid = 98 M^–1^ cm^–1^ and ε_388_ K_4_[Mo(CN)_8_] = 1,460 M^–1^ cm^–1^).

#### Steady-State Kinetic Constants

ABTS, DMP, sinapic acid, guaiacol and K_4_[Mo(CN)_8_] kinetic constants were estimated in sodium phosphate/citrate buffer (pH 4.0, 100 mM). Reactions were performed in triplicate, and substrate oxidations were followed through at the corresponding maximum wavelengths of each reaction product (ε_418_ABTS^∙+^ = 36,000 M^–1^ cm^–1^; ε_469_ DMP = 27,500 M^–1^ cm^–1^; ε_512_ Sinapic acid = 14,066 M^–1^ cm^–1^; ε_470_ Guaiacol = 12,100 M^–1^ cm^–1^ and ε_388_ K_4_[Mo(CN)_8_] = 1,460 M^–1^ cm^–1^). To calculate the *K*_m_ and *k*_cat_ values, the average *V*_*max*_ was plotted against substrate concentration and fitted to a single rectangular hyperbola function with SigmaPlot 10.0, where parameter *a* was equal to *k*_cat_ and parameter *b* was equal to *K*_m_.

### Protein Modeling

The structural model of wild-type PM1Lac at a resolution of 2.49 Å (1 Å = 0.1 nm) (PDB code 5ANH) was used to map the mutations using PyMOL Molecular Graphics System (Version 1.6 Schrödinger, LLC).

### DNA Sequencing

Plasmids containing HRPLs variants were sequenced by GATC-Biotech. The samples were prepared with 5 μL of 100 ng/μL plasmid and 5 μL of 5 μM of each primer, and were as follows: RMLN (5′-CCTCTATACTTTAACGTCAAGG-3′); FS (5′-ACGACTTCCAGGTCCCTGACCAAGC-3′); RS (5′-TCAATGTCCGCGTTCGCAGGGA-3′) and RMLC (5′-GGGAGGGCGTGAATGTAAGC-3′).

## Results and Discussion

### Consensus Design

The departure point of this study was the OB-1 mutant, a HRPL evolved from the basidiomycete PM1 laccase (PM1Lac) (Coll et al., 1993; Mate et al., 2010). OB-1 carries the V[α10]D-N[α23]K-A[α87]T-V162A-H208Y-S224G-A239P-D281E-S426N-A461T mutations that improve its secretion and activity in *S. cerevisiae* (the underlined mutations lying in the evolved α-factor prepro-leader). Using PM1Lac as the query sequence, we first generated three different MSAs that were manually curated and analyzed (see section “Materials and Methods” for details). By removing those sequences that were too short or too large, as well as those that were repeated, the original MSAs were reduced to 102, 718, and 147 sequences for MSA1, MSA2, and MSA3, respectively. These MSAs were then subjected to further computational refinement by imposing RE and MI thresholds (Magliery and Regan, 2005; Sullivan et al., 2012). RE provides information about the frequency of an amino acid at a given position, whereas MI estimates the RE between pairs of these amino acids in specific positions (Durani and Magliery, 2013). The consensus sequences for the three MSAs were calculated using a RE metric and these sequences were compared to that of the OB-1 laccase variant, targeting the positions where amino acids differed as ‘*potential*’ consensus mutations. Substitutions with RE values above the average were preselected and only those that were evident in the three MSAs were chosen, establishing 31 consensus mutations above the threshold (Supplementary Figure S3). We used the MI metric to contemplate covariance, obtaining valuable information about possible correlations and anticorrelations that may exist between each pair of positions. Employing these constraints as a filter reduced the 31 consensus mutations to 20 mutations, selecting only those substitutions with a MI ≤ 0.6 and an above average RE value (excluding entries with a RE > 2.4, i.e., nearly invariant) (Sullivan et al., 2012; Durani and Magliery, 2013; Table 1).

**TABLE 1:** List of consensus mutations that fulfilled the RE and MI thresholds and the comparison with the ancestral node.

Mutation	RE	MI	Consensus/ancestor
I25V	1.80	0.54	V/V
V27A	1.43	0.47	V/A
S33G	2.37	0.22	G/G
D50N	1.28	0.34	N/N
M52L	2.12	0.14	L/L
A92S	1.70	0.42	S/S
D142E	1.26	0.42	E/E
T179G	1.59	0.53	G/G
Y208F	1.17	0.42	F/F
L217M	1.58	0.42	M/M
V219I	1.26	0.28	I/I
Q230L	0.85	0.51	L/L
A240G	1.47	0.27	G/G
D250N	1.59	0.42	N/N
N268G	1.40	0.58	G/N
T306P	2.40	0.30	P/P
M327L	1.47	0.43	L/L
Y421R	1.59	0.59	R/R
F454W	2.40	0.34	W/W
M464F	1.30	0.29	F/F

Consensus mutations often overlap with ancestral mutations, the latter being detected from ancestral sequence reconstructions (Risso et al., 2018). To identify consensus/ancestral mutations, we compared the set of selected mutations with the inferred ancestral node of all basidiomycete laccases, dating back ∼500 million years to the early Paleozoic period (Eastwood, 2014). With the exception of the original Val27 and Asn268 residues, present in both the OB-1 and ancestral node, the remaining 18 substitutions were ancestral/consensus mutations, Table 1. This data is consistent with earlier results where ancestral sequences are close but not identical to their consensus counterparts (Goldsmith and Tawfik, 2013; Risso et al., 2014; and references therein).

### Biochemical Evaluation of Consensus Mutations

**FIGURE 1:**
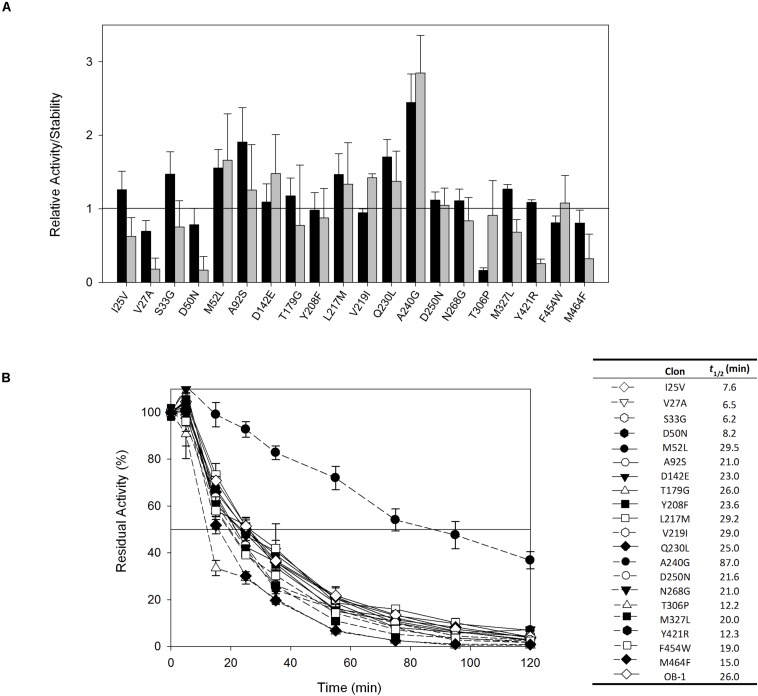
**(A)** Evaluation of the activity and thermostability of consensus variants in terms of the fold-increase. The solid horizontal line represents the activity and thermostability of the OB1 parental type: black bars, initial activity; gray bars, stability given as the ratio between the residual and initial activities. The laccase activity was normalized to the parental activity at each point, indicating the standard deviation from three independent experiments. **(B)** The *t*_1/2_ (in min) at 70°C of the twenty consensus variants and the OB-1 parental type. The solid horizontal line represents the residual activity at 50%. The *t*_1/2_ values and the symbols that pair with each clone are indicated in a table on the right side of the graph. Each point and the standard deviation are from three independent experiments.

**FIGURE 2:**
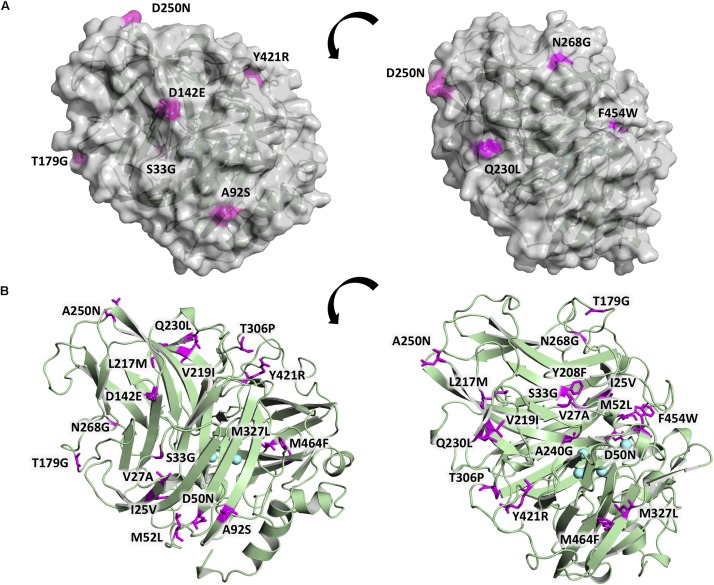
Location of the consensus mutations in the OB-1 variant. **(A)** The laccase surface is shown in transparent gray while the consensus mutations localized at the surface are highlighted in magenta. **(B)** Laccase structure shown as a green cartoon with the consensus mutations highlighted in magenta and labeled, with the coppers depicted as blue spheres. Mutations modeled on PDB ID: 5ANH by Pymol [Schrodinger, LLC (http://www.pymol.org)].

The 20 consensus mutations were inserted individually into the OB-1 laccase, expressed in *S. cerevisiae* and characterized. Most of the clones were as active as OB-1, yet the kinetic thermostability of approximately one-half of the variants was similar to or higher than that of OB-1 (Figure 1 and Supplementary Figure S4). This enhancement is in good agreement with previous consensus designs where roughly 20–40% of the individual consensus mutations had improved thermostability (Lehmann et al., 2002; Komor et al., 2012; Magliery, 2015). As the consensus method tends to break the hypothesized trade-off between activity and stability, consensus mutations are often located far from catalytic sites (Lehmann et al., 2002; Polizzi et al., 2006; Porebski and Buckle, 2016; Siddiqui, 2017). Accordingly, we mapped the consensus mutations in the model of the OB-1 laccase structure and with very few exceptions (see below), they were mostly located far from the catalytic copper centers. Indeed, half of these mutations were found at the protein surface (Figure 2 and Table 2).

**TABLE 2:** Location of consensus mutations.

Mutation	Domain	Secondary structure motif	Relative position	Distance to the T1Cu Site (Å)	Distance to the T2/T3Cu cluster (Å)
I25V	D1	β-sheet	Buried	23.7	15.8
V27A	D1	β-sheet	Buried	25.7	15.6
S33G	D1	Loop	Surface	23.7	14.7
D50N	D1	β-sheet	Partially buried	26.1	18.0
M52L	D1	Loop	Partially buried	24.2	17.6
A92S	D1	β-sheet	Surface	31.8	21.2
D142E	D2	Loop	Surface	29.3	19.7
T179G	D2	Loop	Surface	29.2	29.2
Y208F	D2	β-sheet	Close to Asp205 that interacts with phenols	14.3	13.9
L217M	D2	β-sheet	Partially buried	24.5	22.2
V219I	D2	β-sheet	Buried	23.2	19.1
Q230L	D2	β-sheet	Surface	24.7	23.1
A240G	D2	β-sheet	Close to T2/T3 and His-Cys-His pathway	11.4	5.3
D250N	D2	Loop	Surface	38.0	34.4
N268G	D2	Loop	Surface	23.1	27.0
T306P	D2	Loop	Buried	20.3	25.8
M327L	D3	β-sheet	Partially buried	11.9	13.1
Y421R	D3	Loop	Surface	20.1	13.7
F454W	D3	α-helix	Surface, at the vicinity of the binding pocket	7.3	11.1
M464F	D3	β-sheet	Buried	10.7	17.1

**FIGURE 3:**
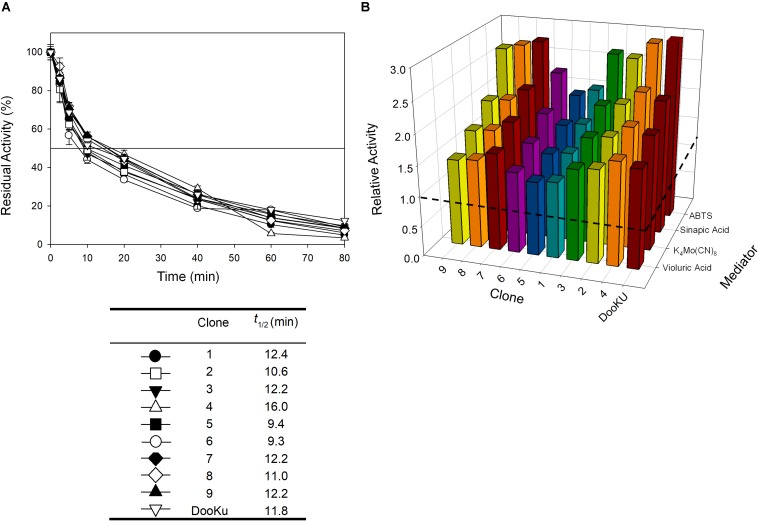
**(A)** The *t*_1/2_ at 75°C of the nine variants from the SDR *in vivo* library and DooKu. The solid horizontal line represents the residual activity at 50% and the *t*_1/2_ and the values of the symbols that pair with each clone are indicated in the table below. Each point (±the standard deviation) is from three independent experiments. **(B)** Total Activity Improvements (TAI) with four different mediators for the variants from SDR *in vivo* library and DooKu. The dashed horizontal line shows the activity of OB-1. Laccase activities were normalized to the OB-1 activity, and each point and standard deviation are derived from three independent experiments.

The recombination of consensus mutations may increase thermostability through a synergistic rather than additive process (Lehmann et al., 2002; Jones et al., 2017). Taking the most stabilizing consensus mutant as a template (A240G -named DooKu), which improved the half-life (*t*_1/2_) at 70°C by 61 min (Figure 1B), we constructed a combinatorial library by site-directed recombination (SDR) *in vivo* (Viña-Gonzalez et al., 2019). Using this method, the six most promising consensus mutations (M52L, T179G, L217M, V219I, Q230L, and D250N) and their corresponding reversions could be rapidly combined on the DooKu template (Supplementary Figure S2A). The SDR library was screened for activity and thermostability using a previously developed high-throughput assay (Garcia-Ruiz et al., 2010). To rule out the presence of false positives, several consecutive rescreenings were carried out (see section “Materials and Methods” for details). After this process, nine variants were selected and characterized (Supplementary Figure S2B). Given that the stable DooKu mutant was the departure template (with a *t*_1/2_ at 70°C of 87 min), to assess kinetic thermostability we enhanced the temperature of incubation by 5°C, measuring the *t*_1/2_ at 75°C (Figure 3A). The ensemble of recombined variants had similar thermostability to DooKu, with slight improvements in some cases like clone 4 that carried the L217M, V219I, D250N, and A240G mutations (Supplementary Figures S2B and Figure 3A). Interestingly, the set of consensus mutations in clone 6 produced a mild decrease in thermostability, which is probably associated with the M52L, T179G, and Q230L mutations that were absent from clone 4 and that exert negative epistasis. The RE and MI values of these substitutions alerted us to potential hidden correlations or modifications that may need to be compensated by other mutations (Durani and Magliery, 2013).

The initial activity was measured with several laccase redox mediators of industrial relevance. In all cases, the same activity pattern was observed (ABTS > sinapic acid > K_4_Mo(CN)_8_ > violuric acid), which indicates a strong correlation between the oxidation rate of laccase and the redox potential (i.e., the higher the redox potential of the mediator compound the lower the oxidation rate) (Figure 3B). Given that the most remarkable contribution to activity and stability came from the single mutation A240G, the DooKu variant and the OB-1 parental type were produced on a larger scale, purified to homogeneity and characterized biochemically (Supplementary Figure S5). The kinetic parameters of the enzymes were evaluated using a selection of redox mediators and phenolic compounds (Table 3 and Supplementary Figure S6). Irrespective of the substrate, the catalytic efficiency was improved as a consequence of an enhanced *k*_cat_ (i.e., the *k*_cat_ of the DooKu variant for DMP, ABTS and sinapic acid was roughly 2-fold higher than that of OB-1, while this increase was milder in the case of guaiacol and K_4_Mo(CN)_8_). Notably, improved kinetic values were accompanied by a 1.4-fold enhancement in secretion while neither the pH activity profile nor the pH stability was affected by the mutation (Supplementary Figure S7).

**TABLE 3:** Kinetic parameters of OB-1 and DooKu variants.

Substrate	Kinetic constant	OB-1	DooKu
ABTS	*K*_m_ (mM)	0.007 ± 0.0006	0.009 ± 0.0009
	*k*_cat_ (s^–1^)	690.0 ± 20.2	1,328.8 ± 42.3
	*k*_cat_*/K*_m_ (mM^–1^ s^–1^)	106,073.8	140,000.8
DMP	*K*_m_ (mM)	0.15 ± 0.01	0.23 ± 0.01
	*k*_cat_ (s^–1^)	565.0 ± 13	1,129.0 ± 18.4
	*k*_cat_*/K*_m_ (mM^–1^ s^–1^)	3,842.6	5,561.8
Sinapic Acid	*K*_m_ (mM)	0.32 ± 0.02	0.45 ± 0.06
	*k*_cat_ (s^–1^)	741.0 ± 14.1	1,433.06 ± 65.2
	*k*_cat_*/K*_m_ (mM^–1^ s^–1^)	2,286.4	3,184.6
K_4_Mo(CN)_8_	*K*_m_ (mM)	0.81 ± 0.2	0.81 ± 0.1
	*k*_cat_ (s^–1^)	220.5 ± 12.1	252.4 ± 11.5
	*k*_cat_*/K*_m_ (mM^–1^ s^–1^)	271.6	313.2
Guaiacol	*K*_m_ (mM)	2.6 ± 0.4	3.4 ± 0.4
	*k*_cat_ (s^–1^)	117.4 ± 6.2	171.0 ± 7.0
	*k*_cat_*/K*_m_ (mM^–1^ s^–1^)	44.6	50.3

*Kinetic constants were estimated in 100 mM phosphate buffer pH 4.0 and 25°C. All reactions were performed in triplicate.*

**FIGURE 4:**
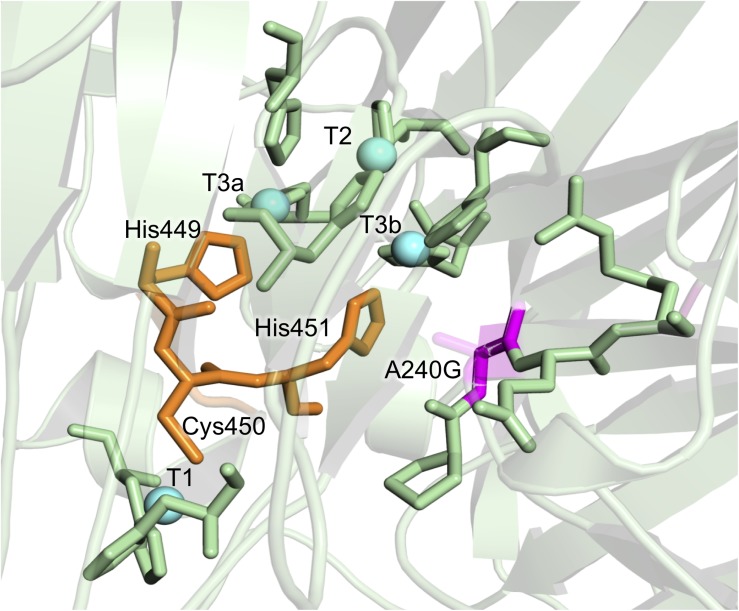
Detail of the A240G mutation from the DooKu variant in the region of the trinuclear Cu cluster. Mutation A240G is shown in magenta with the degraded area corresponding to the lateral chain of Ala240. The copper atoms are labeled and marked as blue spheres, while the residues involved in the electron transfer from the T1 to the T2/T3 are in orange. Residues involved in the first coordination sphere of the catalytic coppers are also represented and the mutations are modeled on PDB ID: 5ANH by Pymol [Schrodinger, LLC (http://www.pymol.org)].

Consensus mutations that improved both stability and activity are rare, since most consensus methods assign low scores to positions around the catalytic core (Goldenzweig et al., 2016; Shivange et al., 2016). In the consensus design used in this study, the relaxed RE/MI thresholds allowed us to identify a highly stabilizing consensus/ancestral mutation located in the surroundings of the catalytic copper T2/T3 cluster for the reduction of O_2_ to H_2_O. The single A240G change produced a noticeable improvement in thermostability, kinetic parameters and expression, even though the substitution of Ala by Gly did not imply any disruption or formation of an H-bond (Figure 4). Indeed, this substitution is roughly located 12 Å away from the T1Cu site, where it takes the binding of the reducing substrate. As such, it is difficult to find a reasonable explanation of the effect of this mutation on the kinetic parameters. We can only speculate that A240G may alter the electron transfer pathway between the T1Cu and the T2/T3Cu cluster, but only a detailed structural and computational characterization could confirm this hypothesis. Despite the striking biochemical effect of this consensus/ancestor mutation is difficult to rationalize, it does prove how consensus design can rapidly identify residues at hotspots that influence laccase catalysis and function.

In summary, an in-house consensus method was designed that predicted 20 laccase mutations, 18 of which were ancestor mutations. The mutations were inserted into an evolved HRPL and roughly half of the mutations incremented their thermostability, while the rest were neutral and in only a few cases destabilizing. The best variants were shuffled by site directed recombination but unlike other consensus designs, no significant positive epistatic effects were found. However, one single substitution produced a dramatic increase in thermostability, kinetics and secretion. The stabilizing consensus-ancestral mutation identified in this work invites us to combine consensus design with other methods like SCHEMA structure guided recombination (Mateljak et al., 2019b) and focused evolution (Vicente et al., 2020) in a drive toward designing superior, more stable HRPLs.

## Data Availability Statement

The raw data supporting the conclusions of this article will be made available by the authors, without undue reservation, to any qualified researcher.

## Author Contributions

BG-F performed the experimental and computational work and wrote the first draft of the manuscript. VR and JS-R designed and performed the computational ASR experiments. MA conceived the project, supervised its development and wrote the final version of the manuscript.

## Conflict of Interest

The authors declare that the research was conducted in the absence of any commercial or financial relationships that could be construed as a potential conflict of interest.

## Footnotes

1 http://mrbayes.sourceforge.net/

2 http://abacus.gene.ucl.ac.uk./software/paml.html

3 http://www.ebi.ac.uk/goldman-srv/WAG/
