# Supplementary material for: Consensus Design of an Evolved High-Redox Potential Laccase
Source: Front Bioeng Biotechnol. 2020 May 6;8:354. doi: 10.3389/fbioe.2020.00354 (PMC7218104; doi:10.3389/fbioe.2020.00354)
Supplement: Supplementary file 1 [file Table_1.PDF]

SUPPLEMENTARY MATERIAL FOR

Consensus Design of an Evolved High-redox Potential Laccase

Bernardo Gomez-Fernandez[1], Valeria A. Risso[2], Jose M. Sanchez-Ruiz[2] and Miguel Alcalde[1]*

[1]Department of Biocatalysis, Institute of Catalysis, CSIC, Cantoblanco, 28049 Madrid, Spain.

[2]Facultad de Ciencias, Departamento de Química Física, Universidad de Granada, 18071 Granada, Spain.

*Corresponding author: E-mail malcalde@icp.csic.es; Tel. +34 915854806; Fax +34 91 5854760.

Supplementary Material contains 7 supplementary Figures and 2 supplementary tables.

SUPPLEMENTARY FIGURE LEGENDS

Figure S1. Robustness of the reconstructed ancestor as measure of the distribution of posterior probability.

Figure S2. Site directed recombination *in vivo*. a) Primers designed for the PCR amplification of the mutated positions selected (in black) in site-directed recombination (SDR) experiments in order to shuffle the best consensus mutations. **b)** Composition of the mutations for the nine recombined variants. The positions mutated to consensus amino acids are highlighted as green squares.

Figure S3. Relative entropy (RE) distributions for consensus mutations common to the three MSAs. Blue bars, MSA1; Red bars, MSA2; Green bars, MSA3. Despite the different treatment and origin of the sequences in the MSAs, the RE values of the selected mutations were mostly consistent in the three groups of alignments.

Figure S4. Functional distribution of consensus variants. IA (initial activity); RA/IA (residual activity/initial activity). Improvements are given in fold *vs*. OB-1 parental type. The A240G DooKu mutant is highlighted in blue.

Figure S5. SDS-PAGE of purified OB-1 parental type and Dooku mutant. Lanes: 1, protein ladder; 2, concentrated crude extract; 3, OB-1 parental type; 4, DooKu mutant.

Figure S6. Kinetic parameters of OB-1 parental type and DooKu mutant fitted to Michaelis-Menten equation for a) ABTS; **b)** DMP; **c)** Sinapic acid; **d)** $K_4[Mo(CN)_8]$ and **e)** Guaiacol. Black circles, OB-1 parental-type; White circles, DooKu mutant. The resulting kinetic parameters are reported in **Table 3**. Each point and the standard deviation are from three independent experiments.

Figure S7. pH stability of **a)** the parental OB-1 and **b)** the DooKu mutant. Laccases were incubated for 0, 4, 24, 48, 72 and 144 hours at different pHs, ranging from 2 to 9, and the laccase activity was normalized to the highest activity value at time 0. Each point and standard deviation is from three independent measurements. **c), d)** pH activity

profile. Black circles, clone 1; White circles, clone 2; Black triangles (down), clone 3; White triangles (up), clone 4; Black squares, clone 5; White squares, clone 6; Black diamonds, clone 7; White diamonds, clone 8; Black triangles (up), clone 9; White triangles (down), OB-1; Black circles in dashed lines, DooKu. Activities were measured with ABTS **c)** or DMP **d)** as the substrate, and normalized to the optimum activity value. Each point and standard deviation come from three independent experiments.

Figure S1

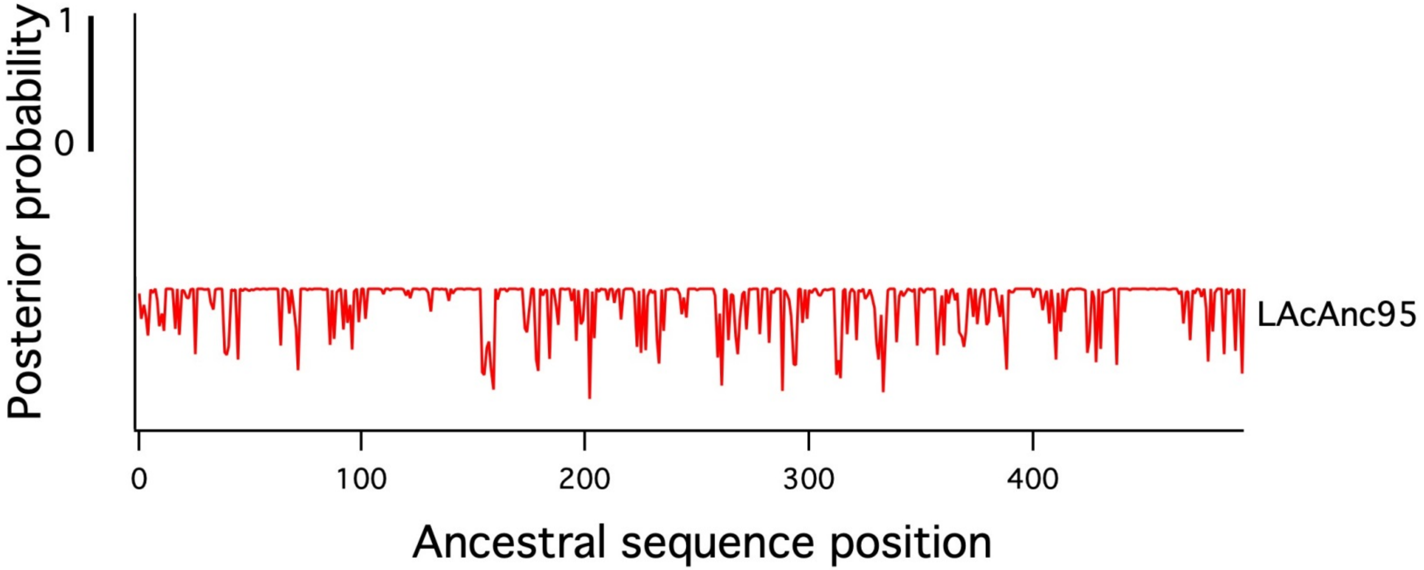
Posterior probability
1
0
LAcAnc95
0
100
200
300
400
Ancestral sequence position

Figure S2

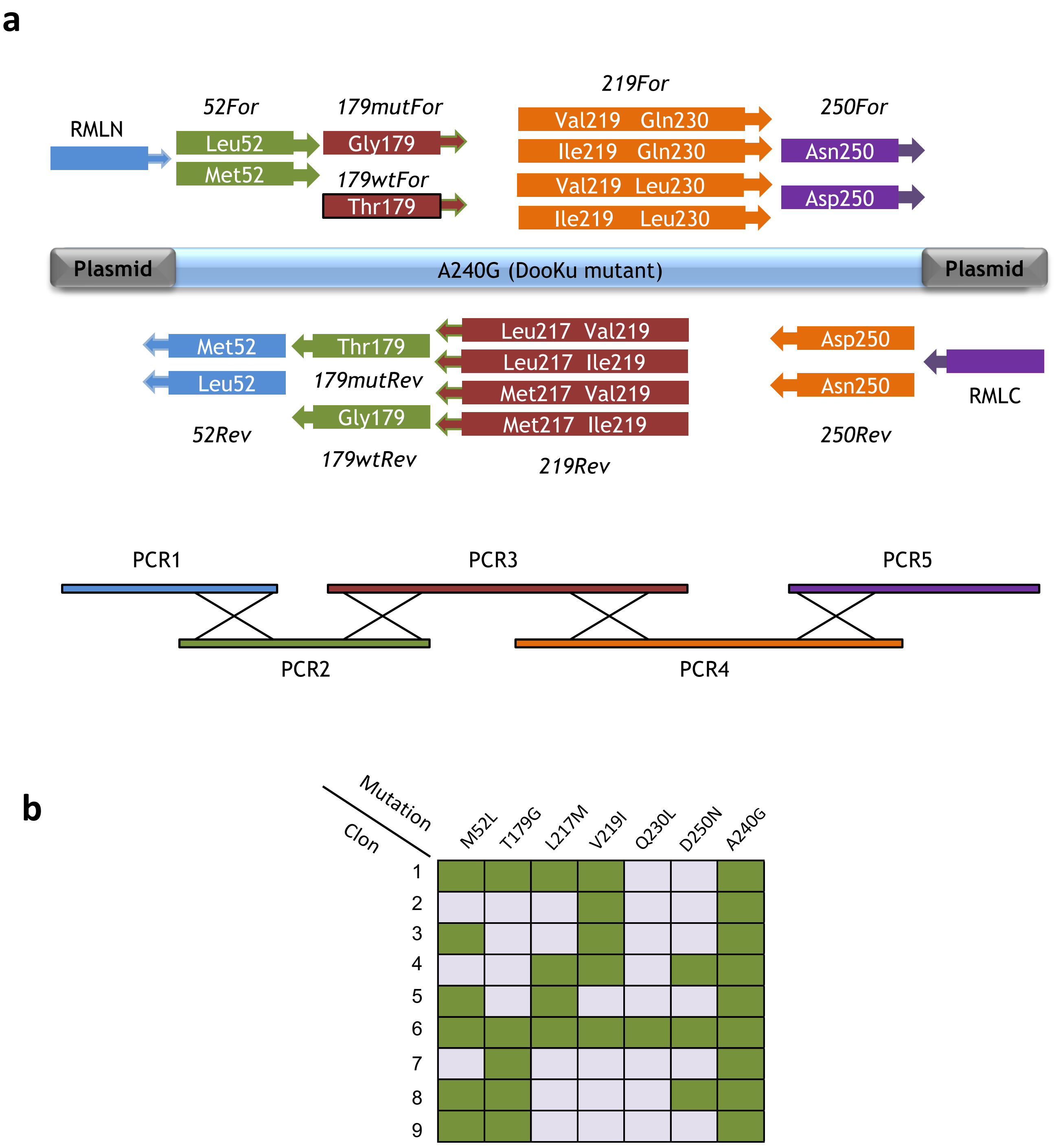
a
RMLN
52For
Leu52
Met52
179mutFor
Gly179
179wtFor
Thr179
219For
Val219 Gln230
Ile219 Gln230
Val219 Leu230
Ile219 Leu230
250For
Asn250
Asp250
Plasmid
A240G (DooKu mutant)
Plasmid
Met52
Leu52
52Rev
Thr179
179mutRev
Gly179
179wtRev
Leu217 Val219
Leu217 Ile219
Met217 Val219
Met217 Ile219
219Rev
Asp250
Asn250
250Rev
RMLC
PCR1
PCR3
PCR5
PCR2
PCR4
b
Mutation
Clon
M52L
T179G
L217M
V219I
Q230L
D250N
A240G
1
2
3
4
5
6
7
8
9

Figure S3

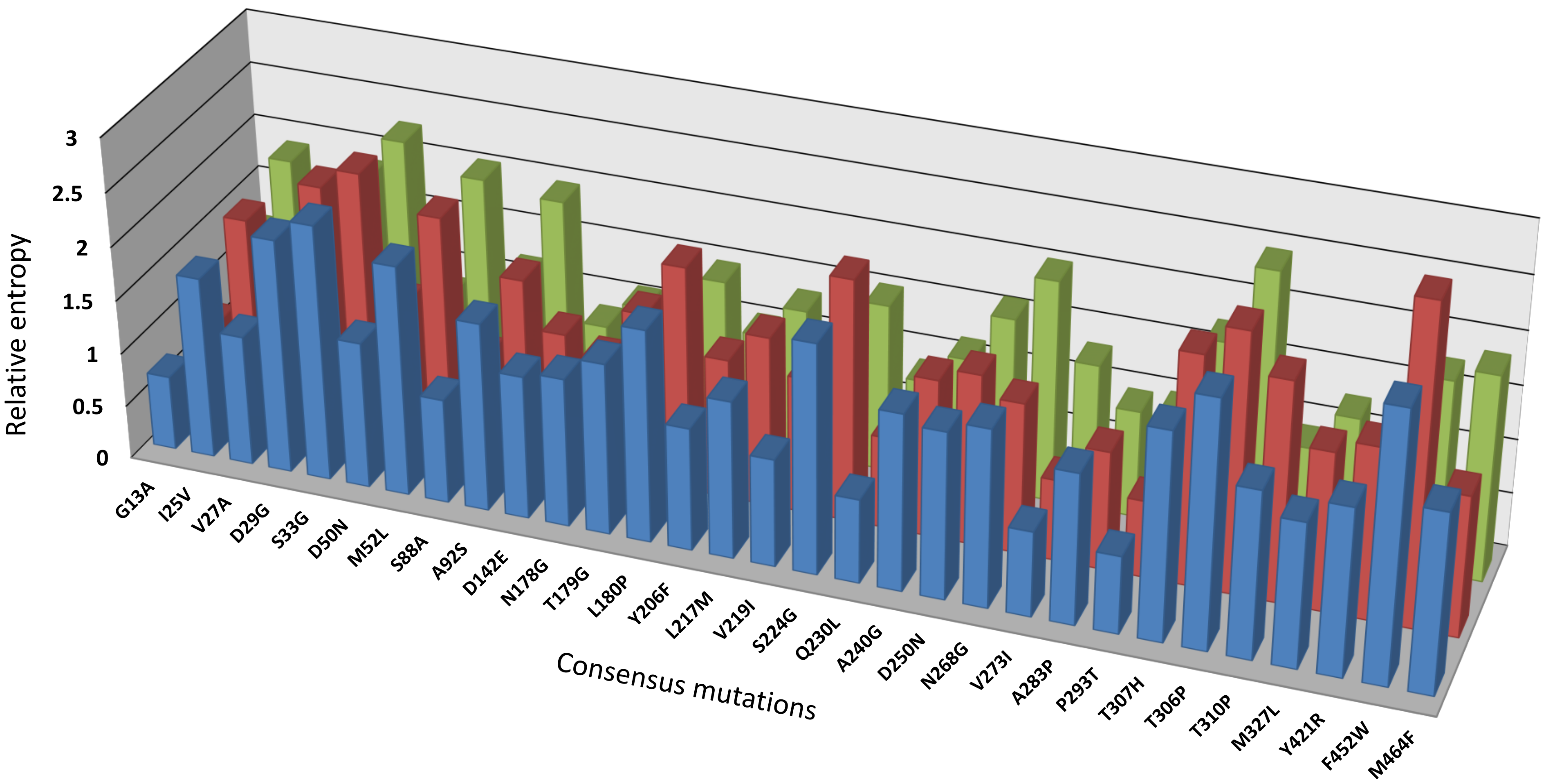

Relative entropy
0
0.5
1
1.5
2
2.5
3
Consensus mutations
G13A
I25V
V27A
D29G
S33G
D50N
M52L
S88A
A92S
D142E
N178G
T179G
L180P
Y206F
L217M
V219I
S224G
Q230L
A240G
D250N
N268G
V273I
A283P
P293T
T307H
T306P
T310P
M327L
Y421R
F452W
M464F

Figure S4

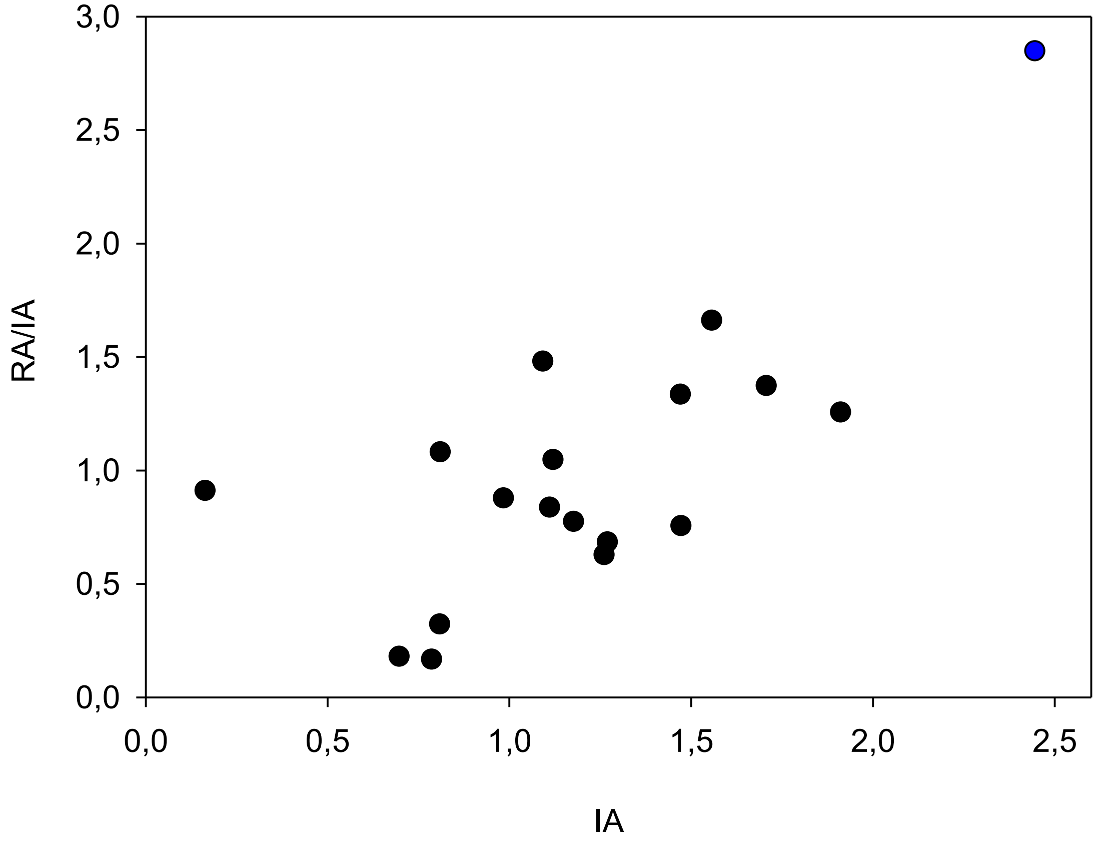

Figure S5

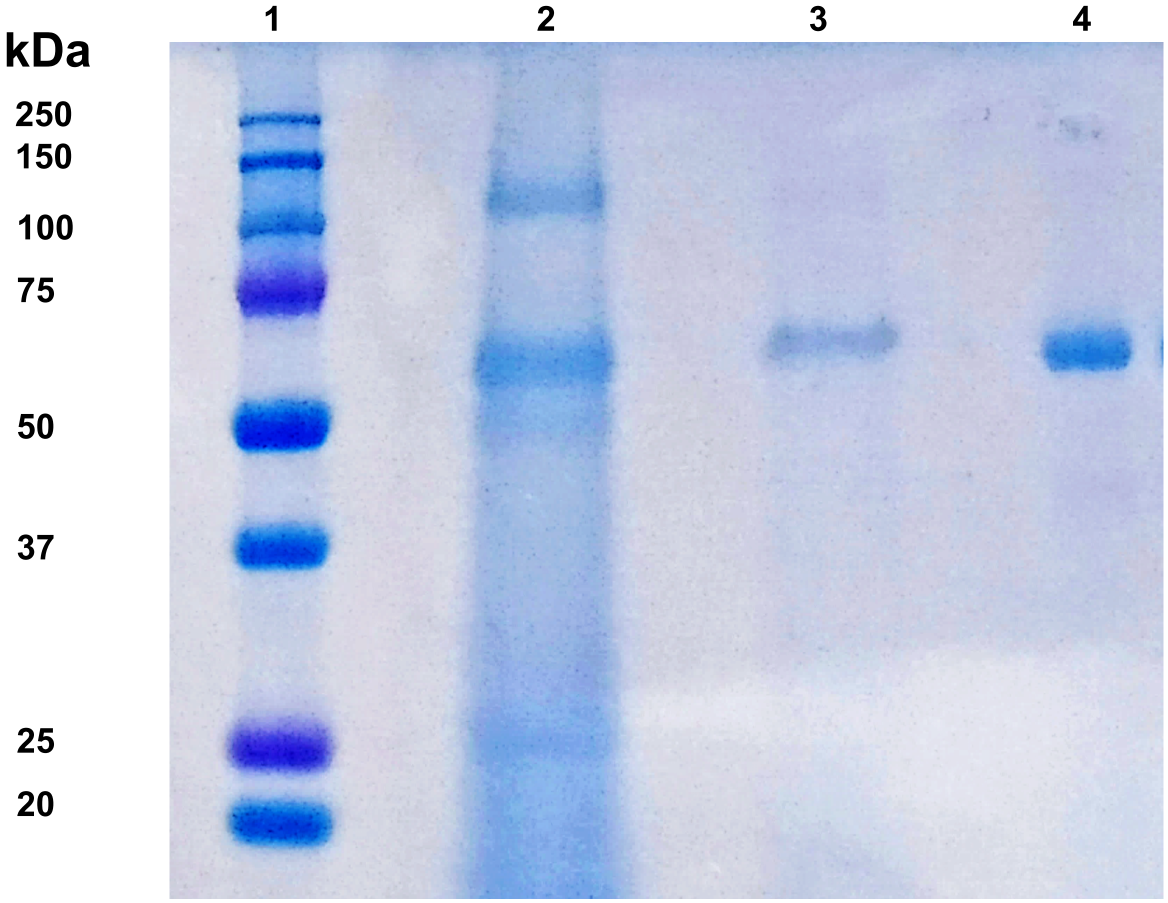

Figure S6

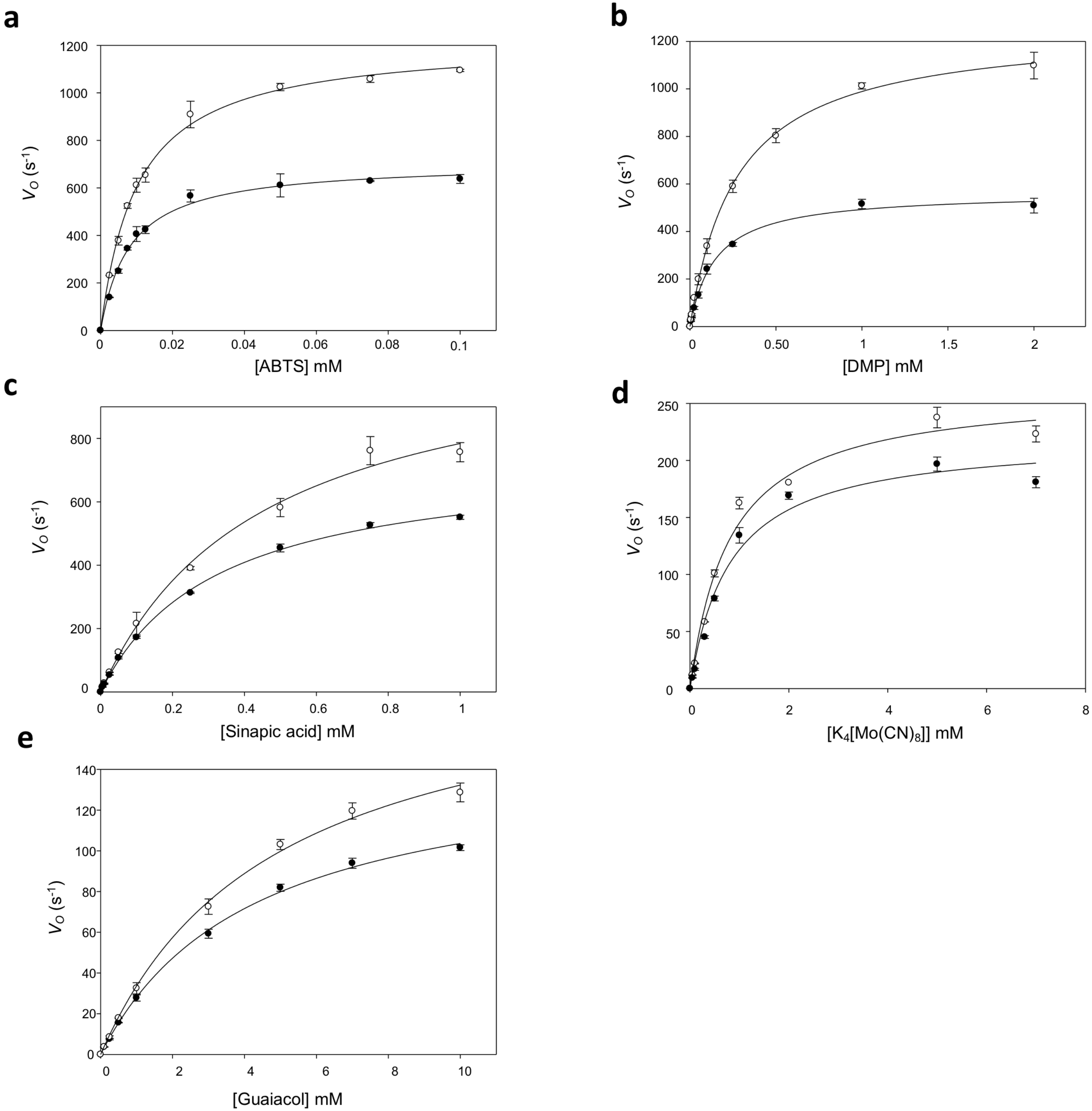
a
b
c
d
e
V_O (s^{-1})
[ABTS] mM
[DMP] mM
[Sinapic acid] mM
[K_4[Mo(CN)$_8$]] mM
[Guaiacol] mM

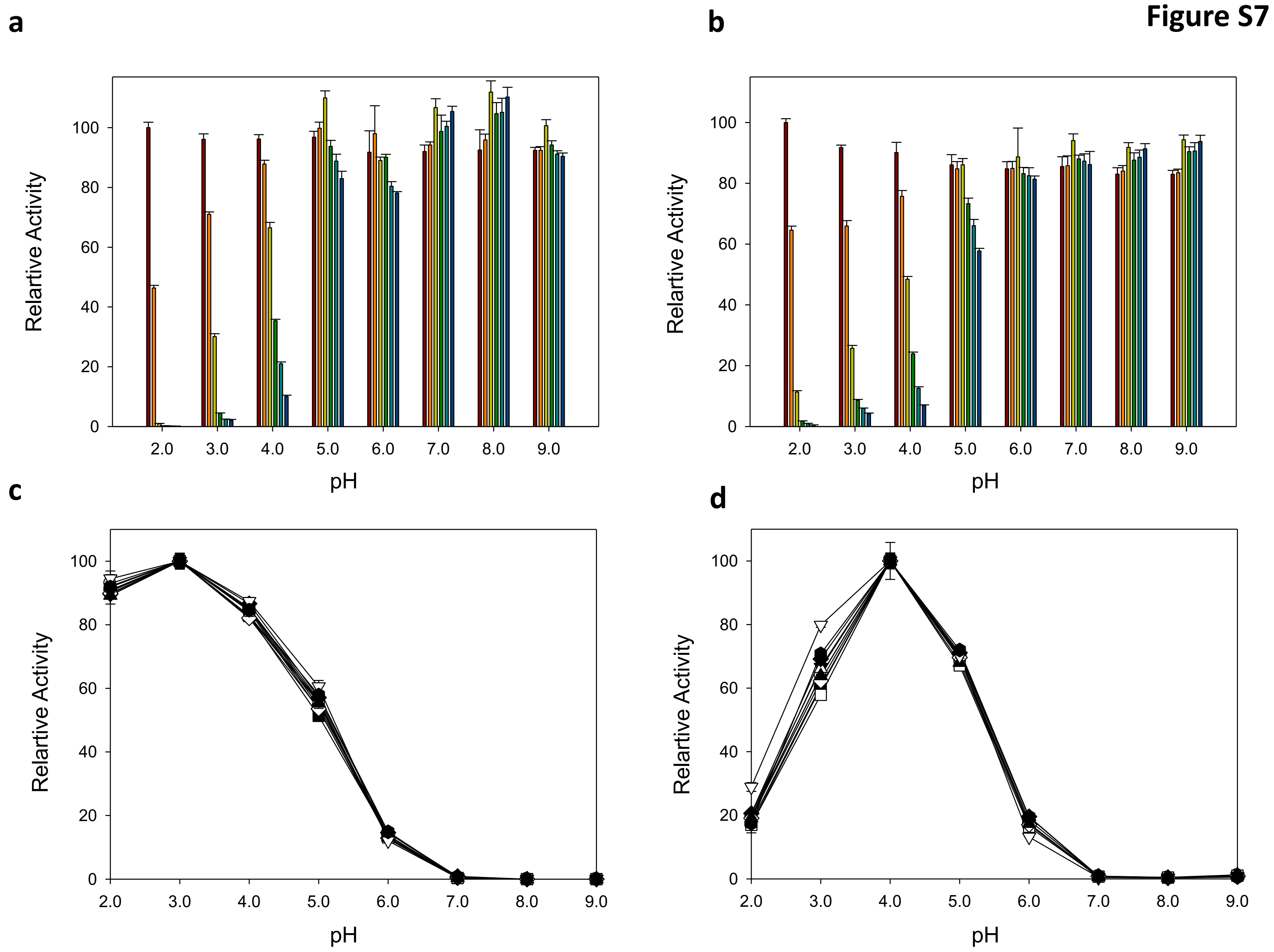

Figure S7
a
b
c
d
Relartive Activity
pH
0
20
40
60
80
100
2.0
3.0
4.0
5.0
6.0
7.0
8.0
9.0

Table S1. Primers for the constructions of 20 consensus mutants

Clone		Primers sequences (5'→3')
I25V	*PCR A*	RMLN: CCTCTATACTTTAACGTCAAGG<br>25R: GGGGAAGACGTCGTTGACCAGGACGGCCTGCCGAGAGAAACCATC
	PCR B	25F: GATGGTTTCTCTCGGCAGGCCGTCCTGGTCAACGACGTCTTCCCC<br>RMLC: GGGAGGGCGTGAATGTAAGC
V27A	*PCR A*	RMLN: CCTCTATACTTTAACGTCAAGG<br>27R : GGACTGGGGAAGACGTCGTTGGCCAGGATGGCCTGCCGAGAGA
	PCR B	27F: TCTCTCGGCAGGCCATCCTGGCCAACGACGTCTTCCCCAGTCC<br>RMLC: GGGAGGGCGTGAATGTAAGC
S33G	*PCR A*	RMLN: CCTCTATACTTTAACGTCAAGG<br>33R: TTGTTCCCCGTAATGAGGGGACCGGGGAAGACGTCGTTGACCAG
	PCR B	33F: CTGGTCAACGACGTCTTCCCCGGTCCCCTCATTACGGGGAACAA<br>RMLC: GGGAGGGCGTGAATGTAAGC
D50N	*PCR A*	RMLN: CCTCTATACTTTAACGTCAAGG<br>50R : ATGGTGTGGTTGGTCATGTTGTTGATGACATTGAGTTGGAAACG
	PCR B	50F: CGTTTCCAACTCAATGTCATCAACAACATGACCAACCACACCAT<br>RMLC: GGGAGGGCGTGAATGTAAGC
M52L	*PCR A*	RMLN: CCTCTATACTTTAACGTCAAGG<br>52R: TTCAACATGGTGTGGTTGGTCAGGTTGTCGATGACATTGAGTTG
	PCR B	52F: CAACTCAATGTCATCGACAACCTGACCAACCACACCATGTTGAA<br>RMLC: GGGAGGGCGTGAATGTAAGC
A92S	*PCR A*	RMLN: CCTCTATACTTTAACGTCAAGG<br>92R : ACCTGGAAGTCGTAAAGGAACGAATGCCCGGTAGAAATCGGGCA
	PCR B	92F: TGCCCGATTTCTACCGGGCATTCGTTCCTTTACGACTTCCAGGT<br>RMLC: GGGAGGGCGTGAATGTAAGC
D142E	*PCR A*	RMLN: CCTCTATACTTTAACGTCAAGG<br>142R : CGCGAGAGTGATTACAGTGGACTCGTCATCAACATCGTAAAGGC
	PCR B	142F: GCCTTTACGATGTTGATGACGAGTCCACTGTAATCACTCTCGCG<br>RMLC: GGGAGGGCGTGAATGTAAGC
T179G	*PCR A*	RMLN: CCTCTATACTTTAACGTCAAGG<br>179R: TGACAGCCAAATCGGCGTTGAGTCCGTTGATGCTGCGACCGAGGCCG
	PCR B	179F: CGGCCTCGGTCGCAGCATCAACGGACTCAACGCCGATTTGGCTGTCA<br>RMLC: GGGAGGGCGTGAATGTAAGC
Y208F	*PCR A*	RMLN: CCTCTATACTTTAACGTCAAGG<br>208R : GAGTGACCATCAATGCTGAACGAATAATTCGGGTCGCATGACAGCG
	PCR B	208F: CGCTGTCATGCGACCCGAATTTCACGTTCAGCATTGATGGTCACTC<br>RMLC: GGGAGGGCGTGAATGTAAGC
L217M	*PCR A*	RMLN: CCTCTATACTTTAACGTCAAGG<br>217R: GCCGTCCGCCTCGATGACGGTCATAGAGTGACCATCAATGCTGAA
	PCR B	217F: TTCAGCATTGATGGTCACTCTATGACCGTCATCGAGGCGGACGGC<br>RMLC: GGGAGGGCGTGAATGTAAGC
V219I	*PCR A*	RMLN: CCTCTATACTTTAACGTCAAGG<br>219R : TTCACGCCGTCCGCCTCGATGATGGTCAGAGAGTGACCATCAAT
	PCR B	219F: ATTGATGGTCACTCTCTGACCATCATCGAGGCGGACGGCGTGAA<br>RMLC: GGGAGGGCGTGAATGTAAGC
Q230L	*PCR A*	RMLN: CCTCTATACTTTAACGTCAAGG<br>230R: GATCTGGATGGAGTCGACAGTCAGGGGCTTGAGATTCACGCCGTC
	PCR B	230F: GACGGCGTGAATCTCAAGCCCCTGACTGTCGACTCCATCCAGATC

		RMLC: GGGAGGGCGTGAATGTAAGC
A240G	*PCR A*	RMLN: CCTCTATACTTTAACGTCAAGG<br>240R : GAGCACAAACGAGTACCGCTGGCCAGGGAAGATCTGGATGGAGT
	PCR B	240F: ACTCCATCCAGATCTTCCCTGGCCAGCGGTACTCGTTTGTGCTC<br>RMLC: GGGAGGGCGTGAATGTAAGC
D250N	*PCR A*	RMLN: CCTCTATACTTTAACGTCAAGG<br>250R : CAGTAGTTGTCCACATCCTGATTTGCGTTGAGCACAAACGAGTA
	PCR B	250F: TACTCGTTTGTGCTCAACGCAAATCAGGATGTGGACAACTACTG<br>RMLC: GGGAGGGCGTGAATGTAAGC
N268G	*PCR A*	RMLN: CCTCTATACTTTAACGTCAAGG<br>268R: GGAGTTAACGCCGCCGTCGAAGCCCCTGGTCCCGGAGTTGGGAAG
	PCR B	268F: CTTCCCAACTCCGGGACCAGGGGCTTCGACGGCGGCGTTAACTCC<br>RMLC: GGGAGGGCGTGAATGTAAGC
T306P	*PCR A*	RMLN: CCTCTATACTTTAACGTCAAGG<br>306R : GGCGCAGCGGTGCCTTCGAGAGGGGTCAGGGCGGACTCCACCAA
	PCR B	306F: TTGGTGGAGTCCGCCCTGACCCCTCTCGAAGGCACCGCTGCGCC<br>RMLC: GGGAGGGCGTGAATGTAAGC
M327L	*PCR A*	RMLN: CCTCTATACTTTAACGTCAAGG<br>327R: CCGCCGGCAAAGCCGAAAGCCAAGTTGAGAGCCAGGTCGACACC
	PCR B	327F: GGTGTCGACCTGGCTCTCAACTTGGCTTTCGGCTTTGCCGGCGG<br>RMLC: GGGAGGGCGTGAATGTAAGC
Y421R	*PCR A*	RMLN: CCTCTATACTTTAACGTCAAGG<br>421R : AGCCCGTGTTGACGACGTCGCCGTAGACCGGGTTCGCGTAGTT
	PCR B	421F: TACAACTACGCGAACCCGGTCCGCCGCGACGTCGTCAACACGGG<br>RMLC: GGGAGGGCGTGAATGTAAGC
F454W	*PCR A*	RMLN: CCTCTATACTTTAACGTCAAGG<br>454R: CGTGAACCCAGCCTCAAGGTGCCAGTCGATGTGGCAGTGGAGGA
	PCR B	454F: TCCTCCACTGCCACATCGACTGGCACCTTGAGGCTGGGTTCACG<br>RMLC: GGGAGGGCGTGAATGTAAGC
M464F	*PCR A*	RMLN: CCTCTATACTTTAACGTCAAGG<br>464R : GACGTCGGGAATGTCCTCGGCGAAGACGACCGTGAACCCAGCCTC
	PCR B	464F: GAGGCTGGGTTCACGGTCGTCTTCGCCGAGGACATTCCCGACGTC<br>RMLC: GGGAGGGCGTGAATGTAAGC

Table S2. Primers used for SDR

PCR		Primers sequences (5'→3')[1]	*Positions*
1	*Primer Forward*	RMLN: CCTCTATACTTTAACGTCAAGG	
	Primer Reverse	*52Rev:* TTCAACATGGTGTGGTTGGT**CAW**GTTGTCGATGACATTGAGTTG	M52/L52
2[2]	*Primer Forward*	*52For:* CAACTCAATGTCATCGACAAC**WTG**ACCAACCACACCATGTTGAA	M52/L52
	Primer Reverse	*179wtRev*: TGACAGCCAAATCGGCGTTGAG**CGT**GTTGATGCTGCGACCGAGGCCG	T179
		179mutRev: TGACAGCCAAATCGGCGTTGAG**TCC**GTTGATGCTGCGACCGAGGCCG	G179
3[2]	*Primer Forward*	179wtFor: CGGCCTCGGTCGCAGCATCAAC**ACG**CTCAACGCCGATTTGGCTGTCA	T179
		179mutFor : CGGCCTCGGTCGCAGCATCAAC**GGA**CTCAACGCCGATTTGGCTGTCA	G179
	Primer Reverse	*217Rev:* CGACAGT**CWG**GGGCTTGAGATTCACGCCGTCCGCCTCGAT**GAY**GGT**CAK**AGAGTGACCA	L217/M217<br>V219/I219 Q230/L230
4	*Primer Forward*	217For: TGGTCACTCT**MTG**ACC**RTC**ATCGAGGCGGACGGCGTGAATCTCAAGCCC**CWG**ACTGTCG	L217/M217<br>V219/I219 Q230/L230
	Primer Reverse	*250Rev:* CAGTAGTTGTCCACATCCTG**ATY**TGCGTTGAGCACAAACGAGTA	D250/N250
5	*Primer Forward*	250For: TACTCGTTTGTGCTCAACGCA**RAT**CAGGATGTGGACAACTACTG	D250/N250
	Primer Reverse	RMLC: GGGAGGGCGTGAATGTAAGC	

[1] Mixed bases are shown in bold (where W= A/T; Y= C/T; K= G/T; R=A/G).

[2] There was no possible degeneration codon for position 179, Thr or Gly, without including other amino acids, therefore a mix of equal quantity of primers for both amino acids were added.
